# Treatment of resistant chronic migraine with anti-CGRP monoclonal antibodies: a systematic review

**DOI:** 10.1186/s40001-022-00716-w

**Published:** 2022-06-04

**Authors:** Hugo Sevivas, Paula Fresco

**Affiliations:** 1 Faculdade de Medicina da Universidade Do Porto (FMUP), Al. Prof. Hernâni Monteiro, 4200 - 319 Porto, Portugal; 2 Laboratório de Farmacologia, Departamento de Ciências Do Medicamento, Faculdade de Farmácia da Universidade Do Porto (FFUP), Porto, Portugal; 3 I3S, Instituto de Investigação E Inovação Em Saúde, Universidade Do Porto, Porto, Portugal

**Keywords:** Resistant chronic migraine, Calcitonin gene-related peptide, Prophylaxis, Anti-CGRP monoclonal antibodies, Erenumab, Galcanezumab, Fremanezumab

## Abstract

**Background:**

Resistant chronic migraine is a highly disabling condition which is very difficult to treat. The majority of the treatments for migraine prophylaxis are nonspecific and present weak safety profiles, leading to low adherence and discontinuation. Currently, monoclonal antibodies (mAb) targeting the trigeminal sensory neuropeptide, calcitonin gene-related peptide (CGRP), are available for migraine prophylaxis being the first drugs developed specifically to target migraine pathogenesis. The main objective of the current work is to carry out a systematic review of randomised controlled trials that specifically analyse the effectivity and safety of anti-CGRP mAb, comparatively to placebo, in patients with resistant chronic migraine and possibly fill the literature gap or be a source of information to health professionals. Additionally the current knowledge on migraine, particularly resistant chronic migraine, was revisited and summarised.

**Methods:**

Literature search was carried out on MEDLINE, Scopus, Science Direct and ClinicalTrials.gov database, from inception to December 2021. Articles were selected according to prespecified criteria of inclusion and exclusion. Efficacy and safety outcomes included were: change from baseline in monthly migraine days (MMD); ≥50% reduction of MMD values from baseline; change from baseline in monthly acute migraine-specific medication days (MAMD); Migraine-specific Quality of Life Questionnaire (MSQ); and registered adverse events. Additionally, we used the Cochrane risk of bias tool (RoB 2) to assess the risk of bias of the included studies.

**Results:**

Four studies were included in this systematic review, involving 2811 resistant chronic migraine patients, 667 in a study using erenumab, 838 in a study using fremanezumab and 1306 in two studies using galcanezumab. When compared to placebo, all investigated anti-CGRP mAb and respective doses demonstrate effectiveness in decreasing MMD, reducing acute medication use and improving the MSQ scores, including, sometimes, reversion of chronic to episodic migraine (efficacy outcomes). Regarding the safety outcomes, the number and type of adverse events did not differ between anti-CGRP mAb-treated and placebo groups.

**Conclusions:**

Anti-CGRP or anti-CGRP receptor monoclonal antibodies are a promising preventive migraine therapy which can be particularly useful for resistant chronic migraine patients.

## Introduction

Migraine is a chronic neurological disorder with paroxysmal features and episodic manifestations characterised by multiphase attacks of head pain associated with other symptoms of neurologic dysfunction, such as sensitivity to movement, photo- and phono-phobia, nausea and vomiting. A migraine attack has three phases: premonitory (prodrome), headache phase and postdrome; each has distinct and sometimes disabling symptoms. About 20–25% of migraine patients have a fourth phase called aura [1]. Migraine can often be recognised by its activators, referred to as triggers. The most common trigger factors are emotional stress, sleep disturbances and dietary factors. Sleep and stress are significant trigger factors in patients with migraine with aura, whereas environmental factors are important trigger factors in patients with migraine without aura. All of them are significant trigger factors in women, contrasting substantially from men [2, 12]. Management strategies involving lifestyle adjustments could be determined by the patient’s susceptibility to specific triggers, although it is becoming recognised that some apparent triggers may in fact be part of the initial phase of the attack, the premonitory phase or prodrome [3].

According to the Global Burden of Disease Study 2016, migraine is the second leading cause of disability and carries significant personal, social and economic burdens [4]. Migraine is the second most prevalent neurological disorder (after tension-type headache), with a female-to-male ratio of 3:1 and an estimated 1-year prevalence of approximately 15% in the general population [5]. The prevalence is higher around the ages of 35 and 39 years and about 3/4 of migraine patients report the beginning of migraine before the age of 35 years [5, 6]. Usually, the condition tends to remit with older age, so an onset of migraine after the age of 50 years should be a warning sign of a secondary headache disorder [5, 7].

Migraine presents itself as three major types, according to the International Classification of Headache Disorders-3 (ICHD-3): migraine with aura, without aura and chronic migraine [8]. Migraine without aura is a recurrent headache disorder exhibiting attacks that last between 4–72 h. Usual characteristics of the headache are unilateral location, pulsating quality, moderate or severe intensity, aggravation by routine physical activity and association with nausea and/or photophobia and phonophobia. Migraine with aura is predominantly characterised by the transitory focal neurological symptoms that usually precede or sometimes are associated with the headache. Some patients also experience a prodromal phase, hours or days before the headache and/or a postdrome phase after headache resolution. Prodromal and postdrome symptoms include hyperactivity, hypoactivity, depression, cravings for particular foods, repetitive yawning, fatigue and neck rigidity and/or pain. The third type is classified as chronic migraine and it is described as a headache occurring on 15 or more days/month for more than 3 months and at least 8 days/month with features of migraine headache (Table 1).

**Table 1:** Diagnostic criteria of migraine according to the ICHD-3 (2018)

Type of migraine	Diagnostic criteria
Migraine without aura	At least five attacks that meet the following four criteria: Headache lasting 4–72 h (when untreated or unsuccessfully treated) Headache with at least two of the following four characteristics: unilateral location; Pulsating quality; moderate or severe pain intensity; aggravation by or causing avoidance of routine physical activity (e.g., walking or climbing stairs) Headache accompanied by at least one of the following symptoms: nausea, vomiting, or both; photophobia and phonophobia Not better accounted for by another ICHD-3 diagnosis
Migraine with aura	At least two attacks that meet the following three criteria: One or more of the following fully reversible aura symptoms: visual; sensory; speech, language, or both; motor; brain stem; retinal At least three of the following six characteristics: at least one aura symptom spreading gradually over a period ≥ 5 min; Two or more aura symptoms occurring in succession; Each aura symptom lasting 5–60 min; at least one unilateral aura symptom; at least one positive aura symptom; headache accompanying the aura or following the aura within 60 min Not better accounted for by another ICHD-3 diagnosis
Chronic migraine	Headaches (suggestive of migraine or tension headaches) on ≥ 15 days/month for > 3 months that fulfil the following criteria: Occurring in a patient who has had at least five attacks meeting the criteria for migraine without aura or the criteria for migraine with aura or both On ≥ 8 days/month for > 3 months, features of migraine without aura or of migraine with aura or believed by the patient to be migraine at onset that is relieved by a triptan or ergot derivative Not better accounted for by another ICHD-3 diagnosis

Adapted from ICHD-3 [8]

## Pathophysiology

Although the pathophysiology of migraine is still, at this date, not completely understood, several recent exhaustive reviews have gathered and explained the multifactorial causes of this neurologic condition [78–80]. Such causes include genetic, anatomical and physiological (neurovascular) alterations, being the bases for the numerous theories that try to explain the various phases of migraine [78–80]. In particular, the neurovascular theory correlates the dura mater vessels innervation by fibres from the trigeminal ganglion and the release of inflammatory neuropeptides, such as the calcitonin gene-related peptide (CGRP) with consequent vasodilation, inflammation and initiation of headache [10]. The trigeminovascular system is considered the anatomical and physiological substrate from which nociceptive transmission originates and produces the perception of migraine pain [9]. Migraine initiation depends on activation and sensitisation of first-order trigeminovascular neurons. The afferent fibres of these neurons innervate the meninges (dura mater) and its vessels and project to structures in the central nervous system [10]. This process sensitises and promotes activation of second-order neurons in the brain stem, third-order neurons in the thalamus and finally nociceptive impulses reach the somatosensory and other cortical areas that are implicated in pain perception [9].

The primary sensory trigeminal neurons reach the *nucleus caudalis* in the brainstem, and from there direct to the periaqueductal grey matter, sensory thalamic nuclei and somatosensory cortex [9, 11]. However, other authors suggest that a primary disruption of central pain pathways produces sensitisation, so that normally innocuous sensory input could be misunderstood as signalling pain, a condition called allodynia [9, 11].

Molecules implicated in the origin of a migraine attack have been detected in clinical models of migraine [15], which are potent vasodilators and are usually distributed in the trigeminovascular system, include CGRP, pituitary adenylate cyclase-activating peptide 38 (PACAP-38) and nitric oxide [15]. Studies have confirmed that migraine attacks develop in patients with migraine when they are exposed to these molecules, while healthy persons similarly exposed refer to experience mild or no headache [15–20]. Moreover, a different study has shown that patients with chronic migraine have significantly elevated serum CGRP levels, even without a migraine attack, when compared with healthy controls [43].

A fundamental characteristic of migraine is its recurrent nature. Patients frequently refer to factors that they identify as triggering their migraine attacks (stress, sleep disturbances, particular foods and not eating) [2, 12]. However, self-retrospective evaluations are limited by recall bias and false attribution. In a study that intended to induce migraine attacks by exposing patients to self-perceived triggers, only a limited number of patients had migraine attacks after exposure, indicating that the role of these triggers could be limited [13, 14].

## Chronification

Approximately 2.5%–3% of patients with episodic migraine (EM) progress to chronic migraine (CM) each year [27–29]. Progression, transformation, or chronification occur when migraine attack frequency increases above 15 days with migraine per month [28]. Migraine-associated symptom profiles and headache-related disability normally also increase in this process. Clinical progression is frequently linked with the experience of cutaneous allodynia and sensitisation at the level of the trigeminal *nucleus caudalis* and these are recognised as signs of physiologic progression to chronic migraine [29].

### Comorbidities associated with chronic migraine

Both clinical and population-based studies revealed a higher number of medical and psychiatric comorbidities in persons with chronic migraine (CM) compared to those with episodic migraine (EM) [30–32]. Patients with CM are approximately twice as likely to have depression, anxiety and pain-related comorbidities compared with those with EM [33]. Respiratory disorders including asthma, bronchitis and chronic obstructive pulmonary disease and cardiovascular risk factors, including hypertension, diabetes, high cholesterol and obesity are also substantially more likely to be present in patients with CM [34, 35].

### Risk factors

Risk factors for chronification can be categorised as: nonmodifiable (older age, female sex, Caucasian race, low education level, low socioeconomic status and genetic factors), modifiable (baseline headache frequency, obesity, medication overuse, snoring, stressful life events, depression and anxiety) and presumed/currently being investigated (proinflammatory states and prothrombotic states) [34, 36, 37].

Among potentially modifiable risk factors, strong evidence was found for increased risk of chronification in patients with higher baseline headache frequency, comorbid depression and medication overuse. Moderate evidence was found for obesity, persistent frequent migraine-associated nausea, cutaneous allodynia, snoring and acute migraine treatment efficacy. Moderate evidence was also found for the nonmodifiable risk factors of comorbid asthma and non-cephalic pain [28, 33].

### Resistant/refractory chronic migraine

Despite advances in the management of headache disorders, some patients with chronic migraine do not experience adequate pain relief neither with acute nor prophylactic treatments [21]. This is associated with higher burden and disability for these patients. The terms resistant, refractory and intractable migraine have been used interchangeably to define this particular condition (chronic migraine that does not respond to two to four prophylactic medications) and various classifications have been suggested over time. In a consensus released by the European Headache Federation, in 2014, refractory chronic migraine was defined as any form of migraine that did not respond to appropriate treatment to two to four classes of prophylactic drugs (β-blockers, anticonvulsants, tricyclic antidepressants, botulinum toxin A) or to acute-phase drugs (triptans, dihydroergotamine, NSAIDs or combinations of analgesics) given in adequate doses [38] (Table 2).

**Table 2:** Diagnosis criteria for refractory chronic migraine accepted by the European Headache Federation (2014)

1: Chronic migraine—no overuse of medication
2: Use of prophylactic medication in adequate doses for at least 3 months with each drug
3: Lack of effect (or contraindications for use) of 2 to 4 drugs from each of the following groups after appropriate treatment^#^
A: β-blockers: propranolol, metoprolol, atenolol, bisoprolol
B: Anticonvulsants: sodium valproate, topiramate
C: Tricyclic antidepressants: amitriptyline
D: Others: flunarizine, candesartan
E: Botulinum toxin A
4: Appropriate psychiatric treatment or other comorbidities carried out by a multidisciplinary group, if available

Adapted from [38]

^#^Appropriate treatment is commonly understood as the time during which adequate doses of an indicated medication are administered, typically at least 2 months (preferably three) at the optimal dose or maximum tolerated dose, unless terminated earlier due to side effects. This concept requires the control of the factors promoting chronification [38, 39]

Meanwhile, a new consensus, in 2020, revised this concept and presented only two subgroups for this difficult to treat condition: the resistant and the refractory chronic migraine [39] (Table 3). Resistant migraine is defined when patients suffer from at least 8 debilitating headache days per month for at least 3 consecutive months without improvement after, at least, 3 classes of migraine preventative drugs in appropriate treatment had failed. It is considered to be refractory migraine when patients have tried all the available preventive medications without effect and suffer more than 8 debilitating headache days per month, for at least 6 consecutive months [39]. Triggers and comorbidities which may contribute to resistant or refractory chronic migraine need to be identified and managed before allocating patients to those categories [for detail see 39].

**Table 3:** European Headache Federation consensus on the definitions of resistant and refractory chronic migraine (2020)

Resistant chronic migraine	Refractory chronic migraine
Established diagnosis of: migraine without aura and/or migraine with aura and/or chronic migraine according to ICHD-3 criteria;	Established diagnosis of: migraine without aura and/or migraine with aura and/or chronic migraine according to ICHD-3 criteria;
Debilitating headache^a^ for at least 8 days per month for at least 3 months;	Debilitating headache^a^ for at least 8 days per month for at least 6 months;
Therapeutic failure^b^ and/or contraindication to 3 drug classes with established evidence for migraine prevention, given at an appropriate dose and duration	Therapeutic failure^b^ and/or contraindication to all drug classes with established evidence for migraine prevention, given at an appropriate dose and duration
Drug classes considered for the diagnosis	
1. Antidepressants (amitriptyline, venlafaxine) 2. Antiepileptics (valproate, topiramate) 3. β-blockers: (propranolol, metoprolol, atenolol, timolol) 4. Calcium channel blockers (flunarizine, cinnarizine) 5. Drugs acting on the CGRP pathway (gepants, monoclonal antibodies) 6. Angiotensin-converting enzyme inhibitor (lisinopril) or angiotensin receptor blocker (candesartan) 7. Onabotulinum toxin A 8. Other pharmacologic preventive treatments with established efficacy in migraine (any new developed drug)	

Adapted from [39]

^a^Debilitating headache is defined as a headache causing serious difficulties to conduct activities of daily living, despite the use of pain-relief drugs with established efficacy, at the recommended dose, and taken early during the attack and therapeutic failure of at least two different triptans

^b^Therapeutic failure may include either lack of efficacy or lack of tolerability

### CGRP and targeted therapy

Preclinical data and clinical models of migraine are the basis for the development of targeted therapies. These include drugs targeting CGRP or its receptor [9]. Calcitonin gene-related peptide is a 37-amino acid neuropeptide, discovered in 1983 [62], involved in several physiological processes in humans [19, 44–46]. Functional studies showed that CGRP is a very potent vasodilator of meningeal arteries and arterioles by activating adenylyl cyclase in the vascular smooth muscle cells [63]. GCRP receptors are present on various cell types in the trigeminovascular system and are considered as having important roles in inflammatory and nociceptive processes [47–49]. CGRP is peripherally released after activation of the trigeminovascular system by migraine triggers and acts also on afferent nerve fibres to exacerbate peripheral sensory inputs and sensitised central trigeminal pathways [22]. For more details on the role of CGRP and the trigeminovascular system and other aspects of migraine pathophysiology please see [78, 80, 86]. Small molecule CGRP receptor antagonists, denominated gepants (namely, ubrogepant, atogepant and rimegepant) had demonstrated clinical efficacy for the treatment of migraine [51–53], but its use has been discontinued after reports of serious liver toxicity associated with their frequent use [54–56]. For that reason, recent interest has been given to the investigation of monoclonal antibodies (mAb) targeting the CGRP pathway. These antibodies do not cross the blood–brain barrier, indicating a peripheral site of therapeutic action in migraine for these drugs [64]. Their advantages include: i) being structurally very different from CGRP antagonists; ii) not being metabolised by the liver and iii) presenting long half-life which allows few administrations (one or two) per month [23, 61]. Anti-CGRP antibodies are thought to impair the effects of excessive CGRP while anti-CGRP receptor antibodies block CGRP receptor activation [50]. By doing so, antibodies against both the ligand and receptor would prevent CGRP-induced activation of sensitised central trigeminal pathways, consequently reducing headache frequency over time [64]. However, it is reasonable to expect that, with increased exposure to exogenous antibodies, an increasing probability of development of anti-drug antibodies happens which can result in a potential decrease of therapeutic activity of the monoclonal antibodies over time [23]. Moreover, in case of occurrence of an adverse effect, their long half-life can represent a liability, as stopping the drug will not stop the adverse effect [23]. Clinical trials using monoclonal antibodies targeting the CGRP ligand (fremanezumab [57], galcanezumab [58] and eptinezumab [59]) or its receptor (erenumab [60]) showed results reporting that these drugs have efficacy for the prevention of migraine and present good safety profiles [24–26].

There are previous systematic reviews and meta-analyses on the safety and efficacy of anti-CGRP mAb in migraine prophylaxis [40–42]. However, evidence related to their effects in resistant chronic migraine is scarce. This lack of evidence and, therefore, evidence-based recommendations for the treatment of this condition with anti-CGRP mAb may be impairing the safe and effective management of these patients. Therefore, the main objective of the current work is to carry out a systematic review of randomised control trials (RCTs) that specifically analyse the effectivity and safety of anti-CGRP mAb, comparatively to placebo, in patients with resistant chronic migraine and possibly fill the literature gap or be a source of information to health professionals.

## Methods

This review was conducted in compliance with the Preferred Reporting Items for Systematic Reviews and Meta-Analyses (PRISMA) guidelines [65].

## Review question

PICO strategy (population, intervention, comparison and outcome) was followed and resulted in the following review question:

- Population: adult patients with resistant chronic migraine;
- Intervention: monoclonal antibodies against CGRP;
- Comparison: placebo;
- Outcomes: change from baseline in monthly migraine days (MMD); ≥ 50% reduction from baseline in MMD, change from baseline in monthly acute migraine-specific medication days (MAMD), change from baseline in Migraine-specific Quality of Life questionnaire (MSQ) scores; safety outcomes (main adverse events, serious adverse events and treatment discontinuation due to adverse events).

## Eligibility criteria

Included studies need to comply with the following criteria: (a) patients—individuals with resistant chronic migraine according to the ICHD-3; (b) intervention—galcanezumab or eptinezumab or fremanezumab or erenumab; (c) comparison—placebo; (d) efficacy outcomes—change from baseline in MMD, ≥ 50% reduction from baseline in MMD, change from baseline in MAMD, change from baseline MSQ scores; (e) safety outcomes; (f) study design—randomised placebo-controlled trials (RCTs).

Exclusion criteria included (a) patients with conditions other than resistant chronic migraine; (b) interventional drugs other than anti-CGRP mAb; (c) association of anti-CGRP mAb with other intervention(s); (d) non-randomised human clinical trials and (e) studies not reporting pre-specified efficacy or safety outcomes.

## Information sources

### Literature search

Four databases (MEDLINE, SCOPUS, Science Direct and ClinicalTrials.gov) were screened for the topic in study from inception to December 2021. The search strategy used for the screening of relevant studies was highly sensitive to collect the maximum of studies: [(galcanezumab OR eptinezumab OR fremanezumab OR erenumab)] AND [(chronic migraine)]. No language restriction or other limits were used.

### Study selection

After screening of studies, duplicates were removed, and the remaining studies underwent a two-stage screening process. The first stage involved title and abstract screening. The second stage involved conducting full-text reading to exclude irrelevant trials. Furthermore, reference lists of included studies were screened to consider additional relevant studies. Two authors independently screened the studies.

### Data extraction process

The following three categories of data were extracted from the included studies: (a) baseline characteristics, (b) efficacy and (c) safety outcomes. Baseline characteristics of the studies included: (i) name of the first author; (ii) year of publication; (iii) national clinical trial (NCT) identifier; (iv) number of previous treatment failure sample size; (v) mean age of participants; vi) percentage of females; (vii) MMD at baseline; (viii) MAMD at baseline; (ix) mean time since initial migraine diagnosis (years) and x) MSQ scores. Efficacy outcomes included (i) change in MMD; (ii) variation in MAMD; (iii) proportion and odds ratio *versus* placebo of patients reaching ≥ 50% reduction in MMD from baseline over 3 months and (iv) change in MSQ scores. Safety outcomes included the presence of any type of adverse effect and their grade.

### Risk of bias

The Cochrane Collaboration’s risk of bias tool (RoB 2) was used to assess the risk of bias of the included randomised placebo-controlled trials [66]. This risk tool consists of six domains: (1) randomisation process; (2) deviations from intended interventions; (3) missing outcome data; (4) measurement of the outcome; (5) selection of the reported result and (6) overall bias. Each domain has been scored as: low risk, some concerns or high risk of bias.

## Results

**Fig. 1:**
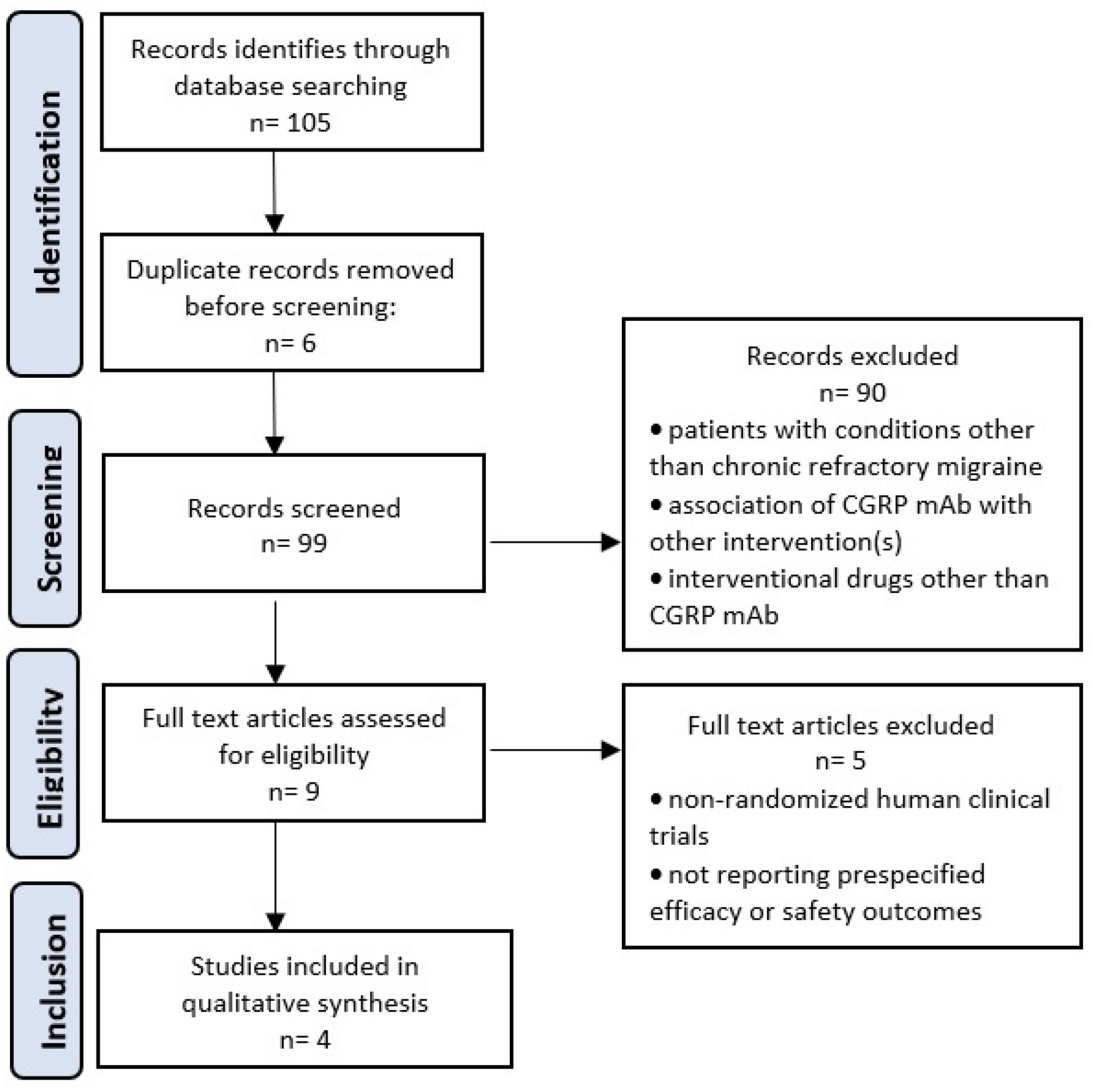
PRISMA flowchart of identification, screening, eligibility and inclusion of studies

Literature search yielded a total of 105 studies. Six duplicate studies were removed before screening. After screening, of the remaining 99 studies, 90 were excluded as they did not meet the inclusion criteria. Full-text screening of the remaining 9 studies resulted in a further elimination of 5 studies that did not match all inclusion criteria or had any exclusion criteria. Finally, only 4 articles were included in the qualitative synthesis (Fig. 1).

The systematic analysis of the literature using the PRISMA [65] described above resulted in four studies that evaluated only three anti-CGRP mAb, namely erenumab, fremanezumab and galcanezumab. These studies collectively included 2811 patients with resistant chronic migraine and evaluated only the effects of erenumab, fremanezumab and galcanezumab.

In the study carried out by Ashina and co-workers [67], the study protocol included monthly administration of placebo or erenumab 70 mg or erenumab 140 mg, for 3 months. In the study carried out by Ferrari and co-workers [68], the study protocol included monthly administration of placebo for 3 months or first dose 675 mg fremanezumab followed matched placebo for 2 months (quarterly treatment) or first dose 675 mg fremanezumab followed by 225 mg for 2 months (monthly treatment). In the study carried out by Ruff and co-workers [69], the study protocol included monthly administration of placebo or galcanezumab 120 mg or galcanezumab 240 mg, for 3 months. In the study carried out by Mulleners and co-workers [70], the study protocol included monthly administration of placebo or galcanezumab 120 mg for 3 months.

The baseline characteristics of the four studies included in this review are comparable between studies and across all subgroups, with similar mean age, MMD, MAMD, years since initial migraine diagnosis, MSQ scores and percentages of female patients (data summarised in Table 4).

**Table 4:** Demographics and baseline characteristics of patients included in the studies used in the final qualitative synthesis

Study	Previous treatment failures	Anti-CGRP mAb	Mean age	N	Female^a^	Mean MMD at baseline	Mean MAMD at baseline	Years since initial migraine diagnosis	MSQ
Ashina et al*.* NCT 02,066,415 [67]	≥ 2	Placebo^b^	42.9 (11.5)	286	78.2%	18.3 (4.5)	11.4 (7.4)	24.0 (12.9)	No data
		Erenumab 70 mg^b^	42.9 (11.2)	191	90.3%	18.0 (4.4)	10.5 (7.2)	25.2 (13.2)	
		Erenumab 140 mg^b^	44.2 (10.6)	190	89.1%	18.8 (4.4)	12.4 (7.2)	24.6 (11.7)	
Ferrari et al*.* FOCUS (NCT03308968) [68]	2–4	Placebo^b^	46.8 (11.1)	279	83%	14.3 (6.1)	12.3 (6.3)	24.3 (13.6)	No data
		Fremanezumab (quarterly treatment)^c^	45.8 (11.0)	276	84.1%	14.1 (5.6)	12.8 (6.2)	24.3 (12.8)	
		Fremanezumab (monthly treatment)^d^	45.9 (11.1)	283	83.5%	14.1 (5.6)	12.2 (6.0)	24.0 (13.7)	
Ruff et al*.* REGAIN (NCT02614261) [69]	≥ 2	Placebo^b^	43.9 (11.8)	558	88.7%	19.6 (4.71)	15.8 (6.0)	24.3 (13.1)	37.5 (17.7)
		Galcanezumab 120 mg^b^	42.8 (11.3)	278	91.9%	20.0 (4.3)	16.6 (5.6)	22.6 (13.3)	39.5 (17.2)
		Galcanezumab 240 mg^b^	42.1 (12.6)	277	82.9%	19.0 (4.9)	14.7 (5.8)	21.3 (13.4)	38.3 (16.9)
Mulleners et al*.* CONQUER (NCT03559257) [70]	2–4	Placebo^b^	44.8 (13.1)	98	87%	18.1 (4.7)	16.4 (6.0)	24.9 (14.9)	40.5 (19.7)
		Galcanezumab 120 mg^b^	45.8 (11.6)	95	87%	19.2 (4.7)	16.0 (6.9)	24.2 (13.9)	41.9 (17.0)

Data are presented as mean (SD) unless stated otherwise

*Anti-CGRP mAb* calcitonin gene-related peptide monoclonal antibodies, *MMD* monthly migraine days, *MAMD*, monthly acute migraine-specific medication days, *MSQ* Migraine-specific Quality of Life Questionnaire, *SD*, standard deviation, *N* number of patients

^a^Percentages represent categorical variables compared to the total number of patients in each treatment subgroup

^b^Monthly administration for 3 months

^c^First dose consists of fremanezumab 675 mg and placebo in the remaining 2 months

^d^First dose consists of fremanezumab 675 mg followed by monthly fremanezumab 225 mg for 2 months

The efficacy outcomes (placebo adjusted mean change from baseline in the number of MMD; proportion of patients reaching ≥ 50% reduction in MMD from baseline and odds ratio *versus* placebo; placebo adjusted mean change from baseline in the number of MAMD and MSQ scores) are summarised in Table 5. The reduction from baseline MMD is significantly higher in the intervention groups compared with placebo groups of the four studies. Statistically significant reductions were also observed in the number of MAMD, in all studies. Additionally, the proportions of participants with a 50% or greater response are also significantly higher in the treatments *versus* placebo groups. The chances of a patient in the intervention groups to have a 50% or great response, are higher than those of patients in the placebo groups, as confirmed by the calculated odds ratio (OR) for each study. Concerning the patients’ quality of life and disability, a significant improvement was also verified from baseline values in the MSQ scores in anti-CGRP mAb-treated patients versus placebo treated patients (in the studies that included this information [68–70]).

**Table 5:** Primary and secondary measured efficacy outcomes in studies included and subgroups in each study, comparatively to those obtained with placebo

Study	Placebo adjusted change from baseline in MMD	Proportion of patients reaching ≥ 50% reduction from baseline in MMD over 3 months	Placebo adjusted change from baseline in MAMD	Placebo adjusted change in MSQ scores
Ashina et al. NCT 02,066,415 [67]	Differences in LSM 70 mg^a, d^: − 2.7 (− 4.2; − 1.2) 140 mg^a, d^: −4.3 (− 5.8; − 2.8) (*p* < 0.001)	Proportion 70 mg: 35.6% 140 mg: 41.3% OR vs placebo 70 mg: 3.5 (1.8; 6.6) 140 mg: 4.2 (2.2; 7.9) (*p* < 0.001)	Differences in LSM 70 mg: − 2.8 (− 3.9; − 1.7) 140 mg: − 4.1 (− 5.3; − 3.0) (*p* < 0.001)	No data
Ferrari et al. FOCUS (NCT03308968) [68]	Differences in LSM Quarterly^b^: − 3.2 (− 4.2; − 2.2) Monthly^c^: − 3.8 (− 4.8; − 2.8) (*p* < 0.001)	Proportion Quarterly 34% Monthly 34% OR vs placebo Quarterly: 5.8 (3.6; 9.6) Monthly: 5.8 (3.6; 9.5) (*p* < 0.001)	Differences in LSM Quarterly: − 3.1 (− 3.8; − 2.4) Monthly: − 3.4 (− 4.0; − 2.7) (*p* < 0.0001)	Difference in LSM Quarterly: 8.8 (5.7; 11.9) Monthly: 10.6 (7.5; 13.7) (*p* < 0.0001; after 4 weeks)
Ruff et al. REGAIN (NCT02614261) [69]	Differences in LSM 120 mg^a, e^: − 4.35 (− 4.52; − 4.16) (*p* < 0.001) 240 mg^a, e^: − 1.77 (− 1.91; − 1.61) (*p* < 0.01)	Proportion 120 mg 29.6% 240 mg 18.7% OR vs placebo 120 mg: 4.05 (2.25; 7.31) (*p* < 0.001) 240 mg: 2.22 (1.26; 3.92) (*p* < 0.01)	Differences in LSM 120 mg: − 4.46 (− 4.64; − 4.28) 240 mg: − 2.06 (− 2.20; − 1.90) (*p* < 0.001)	Differences in LSM 120 mg: 8.45 (7.68; 9.24) 240 mg: 8.57 (7.95; 9.19) (p < 0.01)
Mulleners et al. CONQUER (NCT03559257) [70]	Differences in LSM 120 mg^a, e^: − 3.7 (− 5.2; − 2.2) (*p* < 0.0001)	Proportion 120 mg 32% OR vs placebo 120 mg: 4.8 (2.4; 9.6) (*p* < 0.0001)	Differences in LSM 120 mg: − 3.9 (− 5.3; − 2.4) (*p* < 0.0001)	Differences in LSM 120 mg: 13.9 (8.9; 18.9) (*p* < 0.0001)

Data presented are differences in: least square mean (95% CI), mean percentage, or odds ratio (95% CI)

*LSM* least square mean, *OR* odds ratio, *CI* confidence interval; *MSQ* Migraine-specific Quality of Life Questionnaire, *MMD* monthly migraine days, *MAMD* monthly acute migraine-specific medication days

^a^Monthly administration for 3 months

^b^First dose consists of fremanezumab 675 mg and placebo in the remaining 2 months

^c^First dose consists of fremanezumab 675 mg followed by monthly fremanezumab 225 mg for 2 months

^d^Erenumab

^e^Galcanezumab

The safety outcomes measured in the studies are summarised in Table 6. The incidence of adverse events, serious adverse events and adverse events leading to discontinuation of treatment is comparable between intervention and placebo groups and between the different studies. Nasopharyngitis and injection site-related adverse events, such as pain, erythema and induration were the most common reported incidents. Moreover, serious adverse events and adverse events leading to treatment discontinuation frequencies were not significantly different between the anti-CGRP mAb-treated and placebo groups. No deaths were reported in all studies and authors have considered that none of the serious adverse events observed during the studies were related to the treatments. Only one case of hypersensitivity was reported with galcanezumab in the study carried out by Mulleners and co-workers [70], but no cases of anaphylaxis were reported.

**Table 6:** Safety outcomes measured in the studies included

Study	Main adverse events	Serious adverse events	Adverse events leading to treatment discontinuation
Ashina et al. NCT 02,066,415 [67]	Nasopharyngitis placebo^a^: 5.67% 70 mg^a, d^: 3.16% 140 mg^a, d^: 1.60%	2.5% with placebo (pancreatitis, vomiting, cholecystitis, parotitis, urinary tract infection, intervertebral disc protrusion) 3.2% with 70 mg (non−cardiac chest pain, appendicitis, radius fracture, costochondritis, intervertebral disc protrusion, fibroma) 1.1% with 140 mg (abdominal adhesions, abdominal pain) 0% mortality	0,7% with placebo 0% with 70 mg 1.1% with 140 mg
Ferrari et al. FOCUS (NCT03308968) [68]	Injection−site erythema placebo^a^: 5% quarterly^b^: 7% monthly^c^: 6% Injection site induration 4%, 4%, 5%, respectively^e^ Nasopharyngitis 4%, 5%, 2%, respectively^e^	1% with placebo < 1% with quarterly 1% with monthly Atrial fibrillation, cholelithiasis, clavicle fracture, foot fracture, respiratory fume inhalation, rib fracture, road traffic accident, back pain, nephrolithiasis and vocal cord thickening. None considered treatment related 0% mortality	1% with placebo (chest discomfort, injection−site pain and vulvar cancer) < 1%) with quarterly 1% with monthly (palpitations, fatigue, cholelithiasis, road traffic accidents and temporal arteritis)
Ruff et al. REGAIN (NCT02614261) [69]	Injection site pain placebo^a^: 4.30% 120 mg^a^: 6.23% 240 mg^a^: 7.09% Injection site reaction 2%, 3%, 5%, respectively^f^ Nasopharyngitis 5%, 6%, 3%, respectively^f^	1.25% with placebo (iron deficiency anaemia, myocardial infarction, alcoholic pancreatitis, gastritis, cellulitis, osteomyelitis, epistaxis) 1.83% with 120 mg (cholelithiasis, pyelonephritis, laceration, road traffic accident, colon cancer, squamous cell carcinoma, seizure) 2.84% with 240 mg (acute myocardial infarction, unstable angina, cardiac failure congestive, acute pancreatitis, hypokalaemia, seizure, nephrolithiasis, renal colic, pulmonary embolism, urticaria) 0% mortality	No data
Mulleners et al. CONQUER (NCT03559257) [70]	Injection site reaction placebo^a^: 10% 120 mg^a^: 7% Constipation 2%, 2%, respectively^g^ Nasopharyngitis 9%, 7%, respectively^g^ Influenza 3%, 5%, respectively^g^	1% with placebo (lower limb fracture, Bechet’s syndrome) 1% with 120 mg (haemorrhoids, tonsillitis) 0% mortality	< 1% hypersensitivity reaction

Data are presented as the percentage of patients presenting any adverse effect

^a^Monthly administration for 3 months

^b^First dose consists of fremanezumab 675 mg and placebo in the remaining 2 months

^c^First dose consists of fremanezumab 675 mg followed by monthly fremanezumab 225 mg for 2 months

^d^Erenumab

^e^ Placebo, fremanezumab quarterly treatment, fremanezumab monthly treatment

^f^Placebo, 120 mg galcanezumab, 240 mg galcanezumab

^g^Placebo, galcanezumab 120 mg

**Fig. 2:**
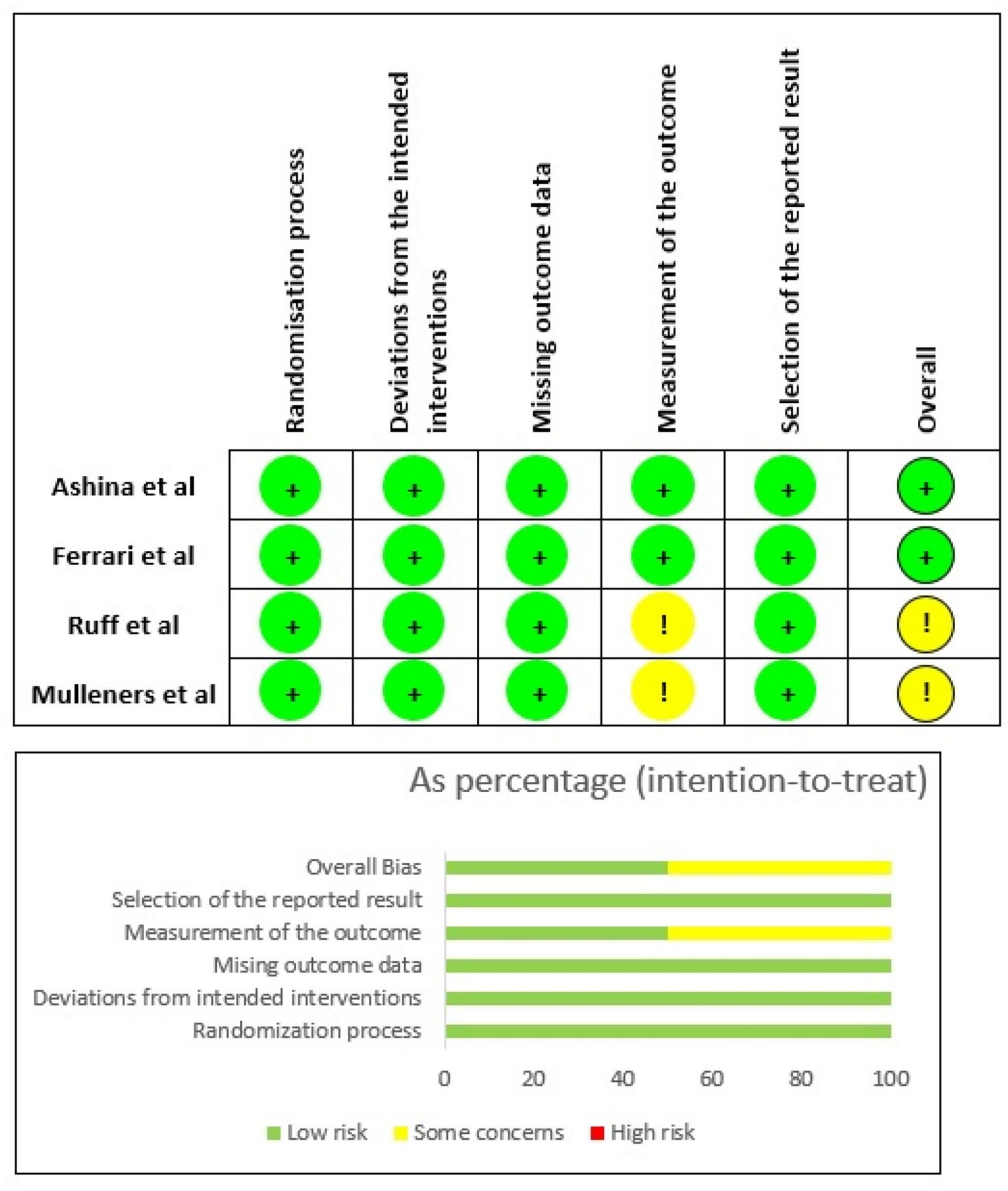
Risk of bias summary and graph obtained using the Cochrane risk of bias tool (RoB 2) [66]. Green represents “low risk” of bias and yellow “some concerns” relatively to risk of bias

The four RCTs included in this synthesis were scored as presenting “low risk” of bias for the following domains of the Cochrane risk of bias tool (RoB 2): randomisation process, deviations from the intended interventions, missing outcome data and selection of the reported results (Fig. 2). However, in two of the studies, the domain measurement of the outcome, did not show enough information to determine whether the outcome evaluators were aware (or not) of the intervention received by the study participants in each arm. Therefore, these were scored as “some concerns” (yellow Fig. 2). Despite that, it is not likely that assessment of the outcome was influenced by knowledge of intervention received. Overall, all included studies showed low-to-moderate risk of bias (Fig. 2).

## Discussion

Clinical trials have suggested that anti-CGRP mAb are an effective treatment to prevent migraine and have some improvements over the treatments used in the past, such as fewer and milder side effects, high target specificity, longer half-life and better patient compliance [23, 61].

The four studies (mainly with low risk of bias and, therefore, good methodologic quality [77]) analysed in the qualitative synthesis of this systematic review included 2811 patients with resistant chronic migraine and used three anti-CGRP mAb (erenumab, galcanezumab and fremanezumab). We could not find any RCT that met the inclusion criteria for eptinezumab. This anti-CGRP mAb is the most recently introduced in the market although it has been already in systematic reviews concerning the effects of all anti-CGRP mAb in prophylaxis of other types of migraine [40–42].

The baseline characteristics of the four studies included in this review are comparable between studies and across all treatment groups, with similar mean age, MMD, MAMD, years since initial migraine diagnosis and MSQ scores (Table 4). These findings make the interpretation of the effects of treatments on each individual study and respective groups easier and more reliable. Moreover, the consistently high percentages of female patients described in all studies are in line with the described epidemiology of this condition [5]. Results comparing efficacy outcomes (MMD, MAMD, ≥ 50% reduction from baseline in MMD and MSQ scores) revealed significantly positive effects of treatments with the three anti-CGRP mAb studied in patients with resistant chronic migraine. These results are in agreement with those from other systematic reviews reporting that anti-CGRP mAb improves prophylaxis of migraine in general [40–42]. The improvements in the measured migraine treatment efficacy outcomes with anti-CGRP mAb compared with placebo in the studies included in the qualitative synthesis were accompanied by improvements in patient reported quality of life scores (MSQ). One possible explanation for these increased MSQ scores can be the fact that the significant reduction in MMD can even be able to revert chronic to episodic migraine (according to the ICHD-3 classification), fulfilling the criteria for this much less debilitating condition at the end of the intervention.

Moreover, the safety outcomes including main adverse events, serious adverse events and adverse events that led to treatment discontinuation are also in line with previously published results [40–42]. These outcomes (adverse events) presented very low frequencies which were similar to those obtained in patients receiving placebo (Table 6). Additionally, these rates were significantly lower and less serious than those reported to occur with traditional migraine prophylactic treatments [82–85].

To the best of our knowledge, this is the first study to collect and discuss data about the efficacy and safety of anti-CGRP mAb in the subgroup of resistant chronic migraine patients, with previous failures to several preventive medications. The decision to include only randomised placebo-controlled clinical trials was made to guarantee high-quality evidence. Moreover, the analysis included subgroup evaluation corresponding to the most used anti-CGRP mAb doses for each molecule to preserve consistency about drug dosing.

The current review presents, however, several limitations. The studies excluded patients with serious or unstable medical conditions such as major cardiovascular conditions, which can limit the extrapolation of the safety results to all patients with chronic resistant migraine. However, treatments with anti-CGRP mAb have shown to be safe in patients with previous cardiovascular risk factors and have not shown significant differences in adverse outcomes, cardiovascular or others, when drug treatment and placebo groups were compared [71]. Moreover, the influence of gender distribution could not be investigated as the trials included predominantly female patients (as expected from the epidemiology of migraine [5]). Another limitation is associated with the small number of participants in each study and the short duration (3 months) of the double-blind period, as well as the limited number of studies included in our review (four RCTs). One possible explanation for the low number of publications found that fulfil the eligibility criteria could be related to the fact that anti-CGRP mAb are a very recent therapy, which has been introduced in the market only a few years ago: the first anti-CGRP mAb was erenumab, in 2018 [60]. This fact can explain the reason for the lack of large studies carried out, either RCTs or real-world evidence studies. Moreover, the fact that our question was only related to a specific small subpopulation of migraineurs (patients with resistant chronic migraine) could also contribute to a somewhat narrow and restricted research question that will lead to a lower number of results [76].

Although RCTs remain the most rigorous scientific method for evaluating the effectiveness of health care interventions, real-world studies (and real-world data) have grown increasingly relevant in the scientific world in recent years [88]. Despite their various limitations, studies using real-world data have the advantage of better representing the group of patients found in daily clinical practice. An example of a real-world study is the work carried out by Barbanti and co-workers that reported that a 48 week long treatment with erenumab granted persistent effectiveness, safety and tolerability in patients with chronic migraine, previously unresponsive to more than 3 preventive treatments [74]. There are other examples of real-world practical clinical studies, like those carried out by Torres-Ferrús and co-workers [90] and by Scheffler and co-workers [91] confirming that anti-CGRP mAb are effective in patients with resistant migraine. Another real-world study carried out in 2021, in Italy, to answer some questions about anti-CGRP mAb (erenumab and galcanezumab) effects on patient´s migraine after one year of treatment, anti-CGRP mAb discontinuation and follow-up during 3 months revealed rather interesting results. Despite the majority of patients with chronic migraine reverted to episodic migraine after one year of treatment, data collected by these authors demonstrated that, in most patients, the therapeutic effect does not persist after anti-CGRP mAb discontinuation. For this reason, anti-CGRP mAb cannot be considered disease-modifying treatments, at least not after a one-year period of use [75]. It would be necessary to observe the clinical course of patients withdrawing the therapy with these biological drugs after longer treatment periods. However, it is important to note that the current recommendations of the European Medicines Agency point to a time period of only 3 months for the treatment with anti-CGRP mAb after which the benefit of treatment should be evaluated and any subsequent decision to continue treatment should be made on a case-by-case basis and the need to further continue treatment should be regularly assessed.

Further studies with larger numbers of patients, adequate time periods to measure differences in long-term complications or adverse events and real‐world data would be required, in the future, to confirm the effectiveness, tolerability and safety for longer periods of treatment and in populations with more comorbidities. Furthermore, a survey for physicians involved in migraine care, carried out by the European Headache Federation with the endorsement of the European Migraine & Headache Alliance, demonstrated the necessity of more evidence regarding the management of these patients as well as clearer guidelines for physicians [92]. Other forms of action such as combined treatments, as presented in the study carried out by Voloshin and co-workers [93], may represent opportunities that should not be neglected and could be important to explain the mechanisms which contribute to drug resistance in migraine. Finally, there are no data about head-to-head comparison between distinct anti-CGRP mAb, so primary studies will also be needed to improve the evidence-based data. During the elaboration of this review, a protocol to perform a systematic review and meta-analysis comparing the effects and safety profile of different monoclonal antibodies in migraine patients has been published, meaning that this issue is getting increasing interest from researchers to create evidence for clinical practice recommendations although these studies, in particular, do not target solely resistant chronic migraine [87].

## Conclusion

Migraine is associated with high disability and, despite being a prevalent disorder, is often underdiagnosed [81]. It is currently accepted that patients with more frequent and severe migraine will benefit from preventive treatments. These treatments may decrease headache frequency, physical and functional disabilities of migraine, improve quality of life and even reduce direct and indirect costs of migraine. Treatment should consider not only the patient’s symptoms, diagnosis and comorbidities, but also the expectations of the patient [78]. The long-term outcomes, including adverse effects, are particularly important to study, even more so for new drugs such as anti-CGRP mAb. These monoclonal antibodies are the first mechanism-based, disease-specific treatment for migraine prevention. These are targeted molecules and, therefore, present superior specificity comparatively to other available preventive migraine medications, and a large number of possible therapeutic applications, including episodic migraine, chronic migraine, medication overuse headache or episodic cluster headache [72, 73, 89].

Anti-CGRP mAb appear to have a great potential for the treatment and reversibility of resistant chronic migraine. In this review, resistant chronic migraine patients showed better ≥ 50% response rates, lower number of days with migraine episodes, lower acute migraine-specific medication use and improved scores of patients reported quality of life. Moreover, in the population of patients with resistant chronic migraine studied in this review, monoclonal antibodies raised against CGRP or CGRP receptors present adverse events and tolerability profiles similar to those of placebo. These positive results obtained in a quite difficult to treat group of patients, who have not benefited from or tolerated several previous standard-of-care treatments, appear significant and exciting. However, longer treatment periods could uncover currently unknown risks. The physiological changes that end an attack and the factors that influence remission or progression to persistent symptoms in migraine over time, are questions that remain unanswered. Therefore, there is the need to carry out additional RCTs with larger populations and real‐world data studies, in a near future, in order to answer these questions and guide the development of disease-modification strategies and determine the importance of these innovative treatments for migraine prevention.

## Data Availability

All data generated or analysed during this study are included in this published article.

## Abbreviations

CGRP mAb: Calcitonin gene-related peptide monoclonal antibodies
CM: Chronic migraine
EM: Episodic migraine
MMD: Monthly migraine days
MAMD: Monthly acute migraine-specific medication days
MSQ: Migraine-specific Quality of Life Questionnaire
SD: Standard deviation
LSM: Least square mean
OR: Odds ratio
CI: Confidence interval
AE: Adverse events
RCTs: Randomised placebo-controlled trials
